# Sex difference in heart failure risk associated with febuxostat and allopurinol in gout patients

**DOI:** 10.3389/fcvm.2022.891606

**Published:** 2022-08-11

**Authors:** Ching-Lan Cheng, Chi-Tai Yen, Chien-Chou Su, Cheng-Han Lee, Chien-Huei Huang, Yea-Huei Kao Yang

**Affiliations:** ^1^School of Pharmacy, Institute of Clinical Pharmacy and Pharmaceutical Sciences, College of Medicine, National Cheng Kung University, Tainan, Taiwan; ^2^Department of Pharmacy, National Cheng Kung University Hospital, College of Medicine, National Cheng Kung University, Tainan, Taiwan; ^3^Health Outcome Research Center, National Cheng Kung University, Tainan, Taiwan; ^4^Department of Nephrology, Ministry of Health and Welfare, Tainan Hospital, Tainan, Taiwan; ^5^Department of Internal Medicine, National Cheng Kung University Hospital, College of Medicine, National Cheng Kung University, Tainan, Taiwan

**Keywords:** heart failure, febuxostat, allopurinol, gout, sex difference, Taiwan

## Abstract

**Background:**

Gout or rapid reduction in serum uric acid level may increase the incidence of heart failure (HF). To compare the risk of HF between febuxostat and allopurinol in gout patients with coexisting cardiovascular (CV) diseases, the varying severity would be likely to confound the risk estimation. Gout and HF are both sex-related diseases, and the risk difference from the urate-lowering agents between women and men remains unknown.

**Aims:**

To evaluate the HF hospitalisations risk of febuxostat and allopurinol in gout patients in real-world settings.

**Methods:**

A population-based cohort enrolled patients with allopurinol or febuxostat initiation from 2011 to 2018. Participants were grouped into, without (low CV risk group) or with (high CV risk group) a history of recent major CV admission. The primary outcome was HF hospitalization. The secondary outcomes were composite CV events, all-cause mortality, and the cause of CV mortality. We used the ‘as-treated' analysis and Cox proportional hazards model after propensity score (PS) matching. Patients were further stratified into men and women to evaluate the gender differences.

**Results:**

Febuxostat users had a significantly higher risk of HF hospitalization than allopurinol users in gout patients either with low CV risk [hazard ratio (HR) 1.39; 95% confidence interval (CI) 1.25–1.55] or high CV risk [HR 1.36; 95% CI 1.22–1.52]. Particularly, women with gout had a higher risk of HF hospitalization than men.

**Conclusion:**

The HF hospitalization risk was highest in gout women with high CV risk and febuxostat use. Monitoring of HF is warranted in these patients.

## Introduction

Heart failure (HF) hospitalisations were reported to occur more frequently than myocardial infarction or stroke in gout patients in previous studies (1). Because uric acid lowering agents would be indicated for gout patients, the risk associated with HF should not be ignored (2, 3). The risk of HF hospitalization event rate was higher in febuxostat users than in allopurinol users (4.3 vs. 3.9%) in the trial of Cardiovascular Safety of Febuxostat or Allopurinol in Patients with Gout and Cardiovascular Morbidities (CARES) (4). However, all patients enrolled in the CARES study had cardiovascular (CV) history but might be different in severity, that would confound the risk of HF hospitalization events.

In addition, results of previous observational studies comparing the HF risk between febuxostat and allopurinol were inconsistent (5–7).

Gout and HF are both considered sex-dependent diseases (8). Earlier studies have shown that hyperuricemia-associated risks of HF, and cardiovascular mortality are both greater in women than in men (9, 10). Some studies showed that women had more sex-specific CV comorbidities than men with HF (11, 12). In addition, women who were hospitalized with CV disease had a higher risk of readmission for heart failure than men. Some prescription drugs were withdrawn from the market due to greater health risks for women than for men (11). It would be critical to assess the sex difference of HF in gout patients receiving uric acid lowing agents.

Therefore, considering patients receiving uric acid lowering agents would pose various severity of CV disease, we stratified the gout patients into low- and high-risk groups to compare the risk difference of HF between febuxostat and allopurinol exposure. We also compared the HF hospitalization risk between febuxostat and allopurinol in female and male gout patients.

## Methods

### Data source

The National Health Insurance Database (NHID) and Cause of Death Data from 2011 to 2018, provided by the Ministry of Health and Welfare, were used for this study. The databases were accessed at the Health and Welfare Data Science Center in Taipei City, Taiwan (13). The NHID is derived from the claims data of Taiwan's national health insurance program, which covers nearly the entire population (23 million people). The NHID includes registries for beneficiaries, ambulatory care claims, inpatient claims, and prescriptions dispensed at pharmacies. Each medical encounter in the claims data contains diagnosis and procedure codes ([International Classification of Diseases, Ninth Edition, Clinical Modification [ICD-9-CM, up to 2015] and Tenth Edition [ICD-10-CM, after 1/1/2016]), and details regarding drug information (e.g., date of prescription, days of supply for all drugs covered by the program). The cause of death data were derived from the death certificates, which included date of birth, sex, date of death, and cause of death (ICD diagnosis codes: ICD-9-CM until 2008 and ICD-10-CM after 2008). These databases can be linked with personal identification numbers to provide details of patient-level information regarding demographics, clinical data, and cause of death information. This study was approved by the Institutional Review Board of the National Cheng Kung University Hospital (IRB certificate number B-EX-107-018). Informed consent from the study participants was waived because patient-level information from the NHID was anonymous.

### Study design and study population

**Figure 1:**
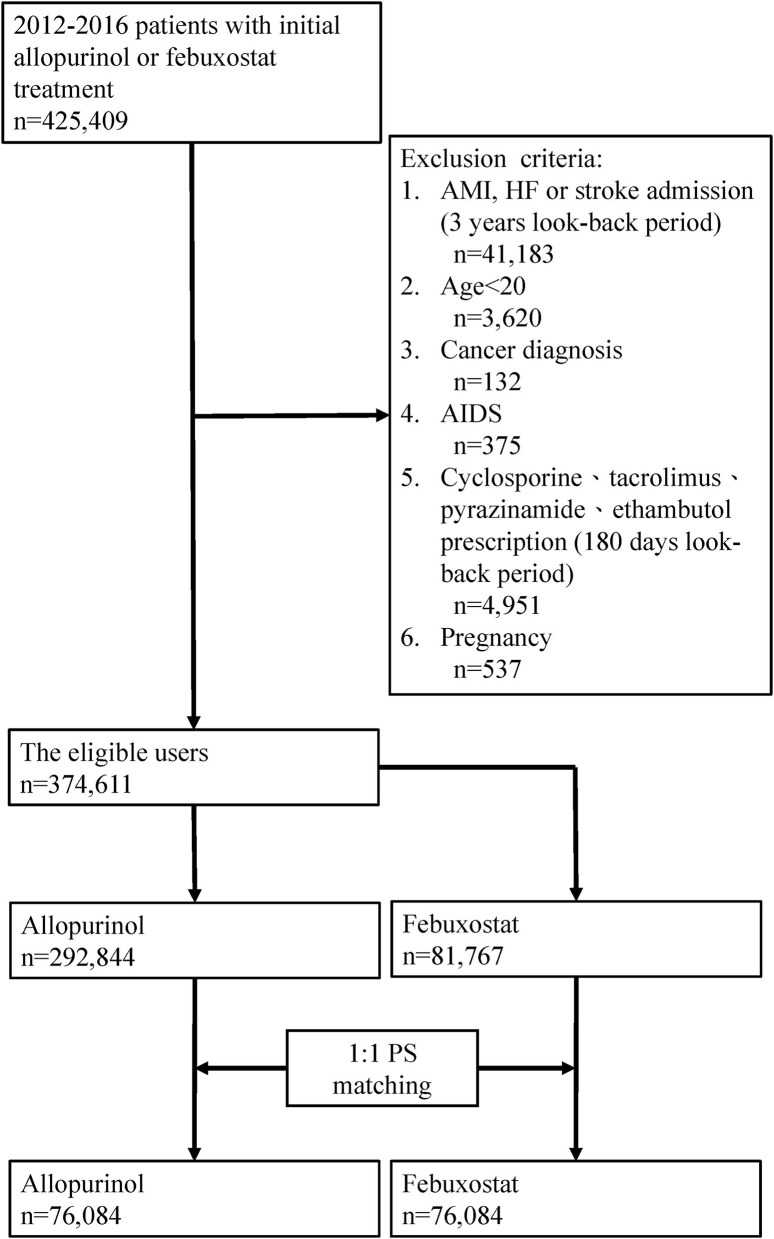
Assembly of the study population.

The present study used a retrospective cohort study with a new user design. We identified a study cohort aged >20 years who had used CV-related drugs and initiated treatment with allopurinol or febuxostat, between 2012 and 2017 (patients had at least one year of follow-up). The CV-related drugs in this study were defined as alpha-blockers, antiplatelets, antithrombotics, antiarrhythmics, beta-blockers, calcium channel blockers, diuretics, or renin-angiotensin–aldosterone system inhibitors. We also estimated nitrates from use with other CV-related drugs. The index date was defined as the date of allopurinol or febuxostat initiation, and a new user was defined as a patient who had not received any febuxostat or allopurinol within 1 year before the index date. Patients were excluded if they met any of the following criteria: (1) cancer history; (2) history of acquired immune deficiency syndrome; (3) pregnancy within the one-year period prior to the index date; (4) prescription of cyclosporine, tacrolimus, rifampicin, pyrazinamide, ethambutol, or isoniazid within 180 days prior to the index date (Figure 1).

### Exposure and high/low CV risk definition

We performed an “as-treated” analysis to estimate whether heart failure occurred within the exposure period. The medication exposure to allopurinol or febuxostat was counted from the date of the first prescription to discontinuation during the study period. Discontinuation was defined as the last day of continuous supply, allowing for a gap of 90 days to account for delayed refills.

We aimed to divide patients into high and low CV risk groups. High CV risk was defined as a history of major CV admission during the 3-year look-back period prior to the index date. Low CV risk was defined as patients who did not have a history of major CV admission during the 3-year look-back period prior to the index date. Major CV admission was defined as patients being admitted for acute myocardial infarction (AMI, ICD-9 code: 410), HF (ICD-9 code: 428), or stroke (ICD-9 code: 430-434 and 436).

### Outcomes, follow-up, and covariates definition

The primary outcome was hospitalization for HF (ICD-9 code: 428). The secondary outcomes were composite CV events, defined as any hospitalization with AMI (ICD-9 code: 410), HF (ICD-9 code: 428), stroke (ICD-9 code: 430–434, and 436), all-cause mortality, and cause of CV mortality. CV mortality was defined as death due to AMI, HF, or stroke. We followed up with patients until the date of one of the following outcomes: death, the end of the study period, or discontinuation of allopurinol or febuxostat, whichever came first. The covariates included in the analyses were age, sex, comorbidities, and concomitant medications. Baseline comorbidities were retrieved during the 1-year look-back period prior to the index date. Patients diagnosed with old myocardial infarction, HF, and stroke from outpatient visits were retrieved as baseline comorbidities. Concomitant medications were retrieved during the 30-day period both before and after the index date. The details of the covariates are shown in Table 1.

**Table 1:** Baseline characteristics of the study population.

**Variable (%)**	**Low CV risk group**			**High CV risk group**		
	**Allopurinol** **(*n* = 61,424)**	**Febuxostat** **(*n* = 61,424)**	**SMD^a^**	**Allopurinol** **(*n* = 12,795)**	**Febuxostat** **(*n* = 12,795)**	**SMD^a^**
**Demographics**						
Sex, male	69.9	69.1	−0.02	64.6	64.3	−0.01
Age, mean (SD)	66.8 (13.6)	66.7 (14.4)	−0.01	72.7 (12.7)	72.6 (13.3)	0.00
Age, %			0.08			0.08
20–29	0.6	0.8		0.2	0.3	
30–39	2.8	3.6		1.1	1.6	
40–49	7.6	8.2		3.7	4.3	
50–59	18.1	16.9		11.3	10.8	
60–69	25.1	24.9		19.6	19.9	
70–79	26.4	24.3		29.4	27.6	
80+	19.4	21.2		34.8	35.7	
**Comorbidities**						
Hypertension	82.4	82.1	−0.01	85.5	85.5	0.00
Gout	51.4	50.4	−0.02	39.2	39.0	0.00
Hyperlipidaemia	48.1	47.1	−0.02	39.4	39.5	0.00
Diabetes mellitus	44.6	44.6	0.00	56.3	56.5	0.01
Osteoarthritis	26.0	26.0	0.00	28.0	28.3	0.01
Ischaemic heart disease	23.8	23.9	0.00	50.1	50.1	0.00
Liver disease	14.1	13.9	−0.01	11.5	11.6	0.00
Kidney disease			0.09			0.04
CKD	36.0	34.2		42.7	41.2	
Pre-ESRD	12.8	14.5		15.2	16.4	
ESRD	2.8	2.6		4.6	4.4	
Stroke	11.8	11.8	0.00	41.9	42.3	0.01
COPD	10.5	10.5	0.00	18.6	18.0	−0.02
Heart failure	9.1	9.4	0.01	54.9	54.4	−0.01
Kidney stone	8.8	8.5	−0.01	5.4	5.2	−0.01
Atrial fibrillation	3.4	3.4	0.00	16.8	16.7	0.00
Peripheral artery disease	2.1	2.0	0.00	3.1	2.9	−0.02
Rheumatoid arthritis	1.9	1.8	−0.01	1.8	1.9	0.00
Acute myocardial infarction	1.0	1.0	0.01	12.7	12.6	0.00
**Co-medications**						
RAAS inhibitors	65.5	64.4	−0.02	62.1	61.3	−0.01
CCBs	45.4	46.1	0.01	48.4	48.6	0.00
NSAIDs	45.4	45.9	0.01	41.8	41.6	0.00
Antidiabetics	40.5	40.3	0.00	52.3	52.4	0.00
Beta blockers	40.2	40.4	0.01	53.0	53.0	0.00
Antiplatelets	38.8	38.5	−0.01	65.2	65.3	0.00
Statins	36.9	36.2	−0.02	38.1	38.4	0.01
Diuretics	35.4	36	0.01	68.3	68.4	0.00
Colchicine	34.5	34.5	0.00	34.6	34.7	0.00
Systemic steroids	24.3	24.6	0.01	33.2	32.5	−0.02
Analgaesics	14.1	14.3	0.01	23.4	23.1	−0.01
Benzbromazone	12.3	12.4	0.00	13.3	13.5	0.01
Nitrates	12.1	12.3	0.01	38.5	37.9	−0.01
Alpha blockers	10.8	11	0.01	13.7	13.7	0.00
Antithrombotics	6.8	7.1	0.01	22.2	22.1	0.00
Antiarrhythmics	4.1	4.2	0.00	12.6	12.3	−0.01
Sulfinpyrazone	1.1	1.1	0.00	1.2	1.3	0.00
Probenecid	0.0	0.0	0.00	0.0	0.0	0.01

a SMD, standardized mean difference.

Low CV risk: No admission for AMI, HF, or stroke prior to the 3-year look-back period.

High CV risk: Admission for AMI, HF, or stroke prior to the 3-year look-back period.

### Statistical analysis

Continuous variables were described as means and standard deviations and categorical variables as numbers and proportions. The distribution of time to event or death since prescription initiation was estimated using the Kaplan-Meier survival analysis. Propensity score (PS) matching was used to adjust for confounding effects. The PS was derived from multiple logistic regression models, and the degree of multicollinearity for all covariates was tested using the PS model. The allopurinol group was matched to the febuxostat group at a 1:1 ratio using a greedy algorithm. The standardized mean difference (SMD) was used to evaluate the degree of different proportions of baseline characteristics between the two groups. In order to consider competing risk, that is, death as a competing risk of CV events, Fine and Gray's sub-distribution hazard model was used (14). For gender analysis, we stratified the study population into male and female groups to estimate the primary and secondary outcomes within low or high CV risk. All statistical analyses were performed using SAS 9.4 version software (SAS Institute, Cary, NC, USA).

### Sensitivity analyses

We conducted two sensitivity analyses to validate the results. First, whether long-term use of allopurinol or febuxostat influences the treatment effect was investigated by including patients who were exposed to allopurinol or febuxostat for at least 1 year and comparing the risk of heart failure, composite endpoints, all-cause mortality, and cause of CV death between allopurinol and febuxostat. Second, serum uric acid level is an important factor associated with the occurrence of CV events and is not available in claim databases. To evaluate the effect of this unmeasured confounding factor, we used the “rule-out approach” based on the method developed by Schneeweiss (15). Two data would be required in this approach, the association between drug use category and confounder (OR_EC_) and the association between confounders and disease outcome (RR_CD_). Then, the apparent relative risk could be calculated to plot the curve within OR_EC_ and RR_CD_.

## Results

The NHID showed that there were 269,580 patients who had used CV-related drugs and had been prescribed allopurinol or febuxostat from 2012 to 2017. After PS matching, 61,424 and 12,795 patients initiated allopurinol/febuxostat at low CV and high CV risk, respectively. The mean age was higher in the high CV risk group than in the low CV risk group. The proportion of male patients was higher than that of female patients in both groups, particularly in gout patients with low CV risk. The baseline characteristics were similar and more comparable in the two groups after PS matching (SMD <0.1) (Table 1).

### Low CV risk group

For the primary outcome, febuxostat users had a significantly higher risk for HF hospitalization than allopurinol users [hazard ratio (HR) 1.39; 95% confidence interval (CI) 1.25–1.55] (Table 2). For secondary outcomes, febuxostat users had a significantly higher risk for composite endpoints than allopurinol users (HR 1.15; 95% CI 1.07–1.24) (Table 2). There was no significant difference between the allopurinol and febuxostat groups in all-cause mortality risk (HR 1.08; 95% CI 0.97–1.19) and cause of CV mortality risk (HR 1.35; 95% CI 0.98–1.85) (Table 2).

**Table 2:** Hazard ratios of heart failure, composite of end points, all-cause mortality, and cause of CV death between patients taking allopurinol and febuxostat.

	**Allopurinol**		**Febuxostat**		**HR (febuxostat vs. allopurinol)**	**95 % CI**	
**Variable**	**No. of event**	**Incidence (per 1,000 person-year**	**No. of event**	**Incidence (per 1,000 person-year**		**Lower**	**Upper**
*Low CV risk group*							
**Primary outcome**							
Heart failure	541	11.4	947	15.8	1.39	1.25	1.55
**Secondary outcome**							
Composite of end points	1,199	25.2	1,744	29.0	1.15	1.07	1.24
All-cause mortality	656	13.8	869	14.5	1.08	0.97	1.19
Cause of CV death	65.0	1.4	104	1.7	1.35	0.98	1.85
*High CV risk group*							
**Primary outcome**							
Heart failure	523	69.2	840	89.2	1.36	1.22	1.52
**Secondary outcome**							
Composite of end points	886	117.2	1,308	138.9	1.26	1.15	1.37
All-cause mortality	309	40.9	425	45.1	1.11	0.95	1.28
Cause of CV death	56	7.4	83	8.8	1.16	0.83	1.64

**Figure 2:**
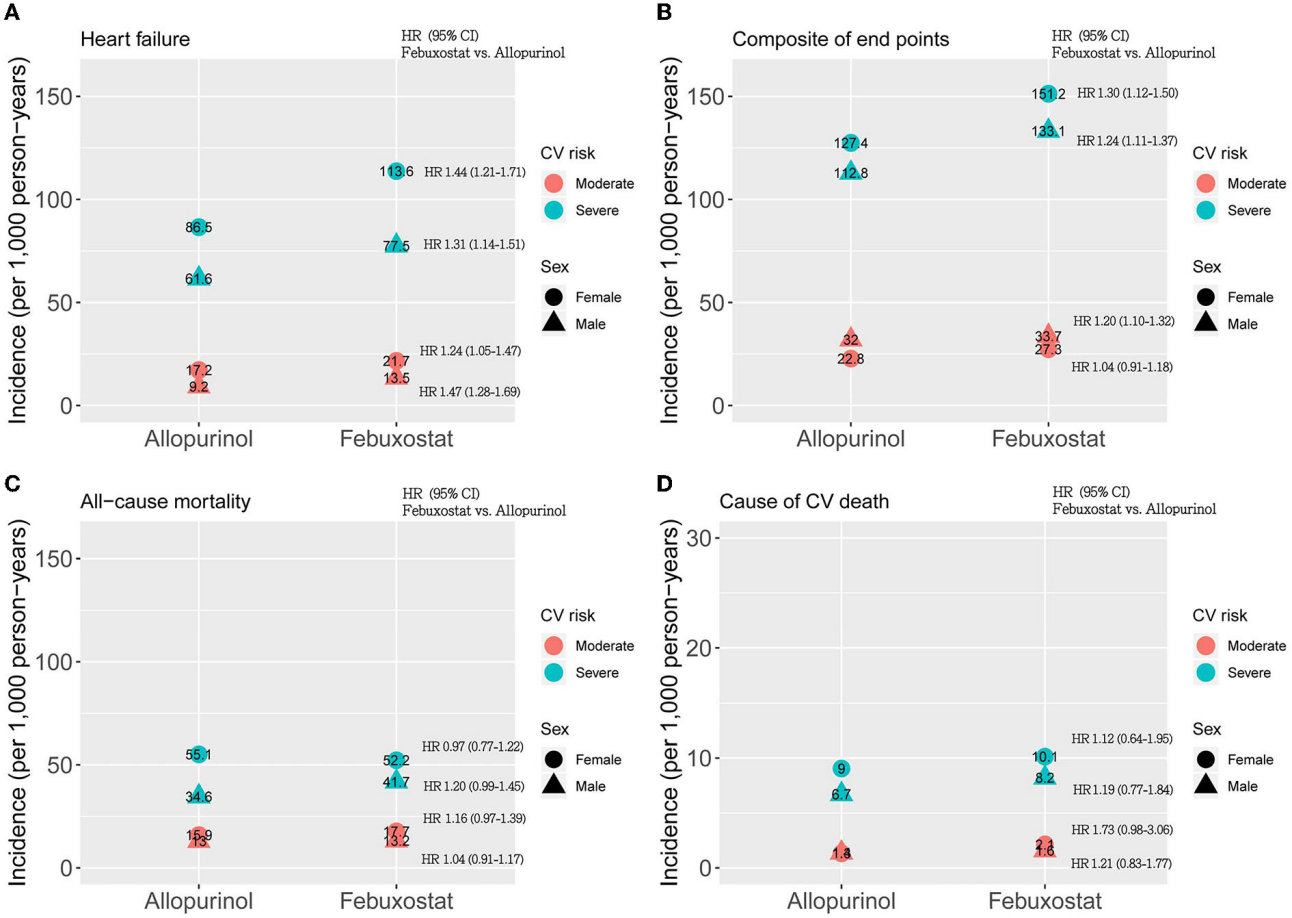
Gender analysis for CV risk-specific hazard ratios of **(A)** heart failure, **(B)** composite of end points, **(C)** all-cause mortality, and **(D)** cause of CV death between patients taking allopurinol and febuxostat. The x-axis shows two drugs: allopurinol and febuxostat, and the y-axis shows the incidence rate per 1,000 person-years. The circles and triangles represent female and male patients, respectively. The red dots represent moderate CV risk, and the blue dots represent severe CV risk. HR represents the hazard ratio. The hazard ratios were estimated in the same strata (the same sex and CV risk groups) for febuxostat users compared to allopurinol users.

Stratified by gender, women (HR 1.24; 95% CI 1.05–1.47) and men (HR 1.47; 95% CI 1.28–1.69) among febuxostat users also had a higher risk of HF hospitalization than allopurinol users (Figure 2). The association of composite endpoint and febuxostat was also found in men (HR 1.20; 95% CI 1.10–1.32) but not in women (HR 1.04; 95% CI 0.91–1.18) (Figure 2).

### High CV risk group

**Figure 3:**
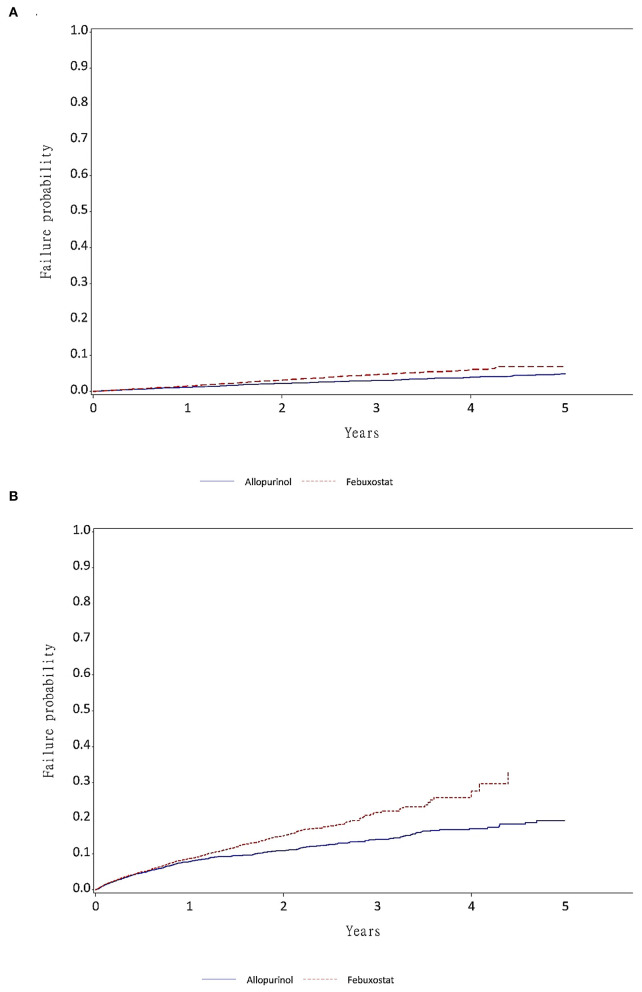
The failure function by year of medication exposure. **(A)** Failure function of time to heart failure in patients without admission for AMI, HF, or stroke prior to the 3-year look-back period (low CV risk group). **(B)** Failure function of time to heart failure in patients admitted for AMI, HF, or stroke prior to the 3-year look-back period (high CV risk group).

The incidence of HF hospitalization was fivefold higher in the high CV risk group than in the low CV risk group (Figure 3). For the primary outcome, the febuxostat users had a higher risk for HF hospitalization than the allopurinol users (HR 1.36; 95% CI 1.22–1.52) (Table 2). With regards to the secondary outcomes, febuxostat users also had a significantly higher risk for composite endpoints than allopurinol users (HR 1.26; 95% CI 1.15–1.37). There was no significant difference between the allopurinol and febuxostat groups in all-cause mortality risk (HR 1.11; 95% CI 0.95–1.28) and cause of CV mortality risk (HR 1.16; 95% CI 0.83–1.64) (Table 2). The primary and secondary outcomes were the same in both women and men (Figure 2).

### Sensitivity analyses

In the sensitivity analysis, we also found that febuxostat had an increased risk of HF hospitalization in gout patients with high CV risk (HR 2.00; 95% CI 1.59–2.51) or low CV risk (HR 1.65; 95% CI 1.40–1.96) for long-term use of medication (Table 3). With regards to the effects of unmeasured confounders (high levels of serum uric acid) in association with allopurinol, febuxostat, and HF hospitalization risk, we found that when the effect of high levels of serum uric acid had a very strong association with allopurinol or febuxostat exposure (odds ratio >10), the observed association of CV risk between allopurinol and febuxostat tended to be negligible (relative risk = 1). However, the chance of a strong association with allopurinol or febuxostat is much lower. Therefore, the effect of high levels of serum uric acid as a confounder might be ruled out because its results were not significant enough to explain this observed association (Supplementary Figures 1A,B).

**Table 3:** Heart failure, composite of end points, all-cause mortality, and cause of CV death between patients taking allopurinol and febuxostat for more than 1-year.

	**Allopurinol**		**Febuxostat**		**HR*a* (febuxostat vs. allopurinol)**	**95% CI**	
**Variable**	**No. of event**	**Incidence (per 1,000 person-year**	**No. of event**	**Incidence (per 1,000 person-year**		**Lower**	**Upper**
*Low CV risk group*							
**Primary outcome**							
Heart failure	218	5.9	407	8.3	1.65	1.4	1.96
**Secondary outcome**							
Composite of end points	480	12.9	713	14.6	1.35	1.19	1.52
All-cause mortality	207	5.6	246	5.0	1.16	0.95	1.4
Cause of CV death	24	0.6	32	0.7	1.45	0.84	2.5
*High CV risk group*					0	0	0
**Primary outcome**							
Heart failure	112	20.6	248	35.4	2.00	1.59	2.51
**Secondary outcome**							
Composite of end points	204	37.6	363	51.8	1.64	1.37	1.95
All-cause mortality	55	10.1	76	10.9	1.26	0.88	1.81
Cause of CV death	7	1.3	18	2.6	2.22	0.90	5.48

^a^HR: The hazard ratios were adjusted by propensity score.

## Discussion

Our study found that febuxostat use had a significantly higher risk of HF hospitalization, but similar mortality compared to allopurinol use in gout patients with low or high CV risk. In gout patients, women were more likely to experience HF hospitalization than men, and the HF hospitalization risk was highest in women with high CV risk and febuxostat use.

Gout is related to a variety of cardiovascular diseases, such as myocardial infarction, stroke, and HF. In the studies in younger populations with mean a age og <50 years old, urate-lowering therapy was associated with lowered coronary artery disease and stroke hospitalization, but not heart failure hospitalization, compared to non-urate lowering therapy in gout patients (16, 17). In particular, gout was associated with an increased risk of HF, rather than an increased incidence of coronary heart disease, stroke, or all-cause mortality, in the REasons for Geographic And Racial Differences in Stroke (REGARDS) cohort study (18). Meanwhile, serum uric acid level was known to be associated with incident cardiovascular events and could be an important prognostic factor for patients with HF (19–21). Animal studies demonstrated that high serum UA increased HF and worse the prognosis, with a possible mechanism *via* its effects on oxidative stress and endothelial dysfunction (22–25). Therefore, it is suggested that lowering uric acid therapy has a beneficial effect on the prognosis of HF (26, 27). However, other studies have reported that individuals whose serum UA was too low might have an increased incidence of cardiovascular disease or mortality (28, 29). In addition, rapid reduction of serum UA levels was associated with readmission of HF (30). In particular, a potent uric acid lowering drug, febuxostat, was found to have higher rates of all-cause mortality and CV mortality than allopurinol in clinical trials (4). In the CARES study, a large and randomized control study enrolled patients with major CV disease. All-cause mortality and cardiovascular mortality were higher with febuxostat use than with allopurinol use. They also found that the HF hospitalization event rate was higher in febuxostat users than in allopurinol users (4.3 vs. 3.9%) (4). The main limitations of the CARES study were the high attrition rate of 45% (lost to follow-up) and high drug discontinuation rate (56%). A population cohort study from Taiwan similarly reported increased adverse CV events in febuxostat users, compared to allopurinol users, and subgroup analysis showed that elevated risk of HF hospitalization (HR, 1.22; 95% CI, 1.13–1.36) (5). However, the mean follow-up duration of the above-mentioned studies were <1 year. In contrast, in a population study using US Medicare claims data with a mean follow-up duration of 1.1–1.2 years, febuxostat users had similar rates of new-onset HF, compared to allopurinol users (6). A trend toward a lower risk for HF hospitalisations in febuxostat users, compared to allopurinol users, was noted in a cohort study from Hong Kong, but the study population was relatively small (7). In the recently published randomized FAST trial, no risk difference of MACEs or HF hospitalisations between febuxostat and allopurinol use was found (31). However, the enrolled population had fewer comorbidities, lower adverse CV event rates, and total mortality than the population from our study, which might not be representative of the real-world setting. Compared to these conflicting results, our national population-based cohort study from the NHID, which covered more than 99.9% of the total population of 23 million in Taiwan with extremely few barriers to medical accessibility, had the advantage of assessing the CV risk of febuxostat in the general population with gout. We identified gout patients with CV risk from the National Health Insurance database by using CV-related drugs, to elucidate the risk of HF hospitalization between febuxostat and allopurinol users in real-world settings. We further divided gout patients into low or high CV risk to evaluate the risk of HF at different CV risk profiles. Moreover, we restricted our analysis to patients, who had received febuxostat or allopurinol treatment for more than 1 year. These analyses yielded consistent results with the elevated HF hospitalization risk of febuxostat, compared to allopurinol. Assuming the follow-up duration was fixed, we estimated the attributable risk of febuxostat in the low and high CV risk groups are 42.87 and 37.7%, respectively. Our study provided valuable information to illustrate the HF hospitalizations risk of febuxostat use as stratified by low or high CV risk in the Asian population.

The mechanism underlying the cardiovascular risk of febuxostat vs. allopurinol was uncertain. Previous evidence has suggested that high levels of uric acid represent an independent CV risk factor and that the use of xanthine oxidase inhibitor (XOI) may reduce the risk of major adverse CV events (MACEs) (32, 33). On the other hand, accumulating evidence suggests that increased xanthine oxidase activity contributes to increased vascular oxidative stress and endothelial dysfunction in HF patients (25). The CV protective effects of allopurinol might be attributed to the anti-oxidant effect by inhibiting the production of reactive oxygen species released during the activity of xanthine oxidase or improving endothelium function along with the reduction of uric acid levels (34, 35). Compared with febuxostat, the CV protective effect of allopurinol might be due to the difference in chemical activities of purine (allopurinol) and non-purine-like (febuxostat) medication and was only seen in patients with allopurinol dosage ≤ 300 mg/day (32). In patients with HF and gout, lower dose allopurinol (≤ 100 mg/day) was found to have reduced HF hospitalizations or death (36). In contrast, in the EXACT-HF study, allopurinol with a target dosage of 300–600 mg/day in heart failure patients failed to improve clinical status (35). This might possibly explain the relatively beneficial effect of lower dose allopurinol in our study.

Our study found that compared to men, women with gout were substantially more likely to experience HF hospitalisations, which was independent of low or high CV risk. This result might be due to the fact that women with gout arthritis had onset of gout at an older age, had increased comorbidities with hypertension or renal insufficiency, and had more frequent use of diuretics (8). Gender differences were observed in the prevalence of heart disease, comorbidities, mortality, and treatment response to HF (11). In the general population, men have a higher incidence of HF, but the overall prevalence rate is similar in both sexes due to better long-term survival after the onset of HF in women (37). HF occurs at an older age and with fewer ischaemic causes in women compared to men. In addition, hypertension and diabetes predispose older women to HF to a greater extent than men (11). In HF patients, some comorbidities were clearly sex-specific, such as arthritis, depression, and hypothyroidism were higher in women, while arrhythmias, ischaemic heart disease, and chronic COPD were higher in men (12). Febuxostat users had an increased risk of HF hospitalisations and similar mortality in both sexes compared to allopurinol users. Interestingly, there was no significant difference between febuxostat and allopurinol users in composite CV outcomes for female gout patients with low CV risk (HR 1.04; 95% CI 0.91–1.18), and further investigation is needed. Therefore, our study reinforced the importance of HF monitoring and management in gout patients, particularly in females and patients taking febuxostat.

Our study had several strengths. First, this was a national population-based cohort study using a claims database to estimate the HF risk of febuxostat compared with allopurinol, adjusted for many known confounders. We also provided safety information regarding febuxostat, which is widely used in Asia where allopurinol hypersensitivity is common (38–40). Second, we divided our patients into low or high CV risk to further analyse the complete risk profiles in general practice. Third, we restricted our analysis to patients with more than 1 year of febuxostat or allopurinol treatment to assess the safety of long-term use. Since febuxostat at usual doses is more potent in lowering uric acid than allopurinol, it is probable that patients who started on febuxostat had higher baseline uric acid than those on allopurinol. Physicians would likely switch allopurinol to febuxostat if uric acid levels were uncontrolled. Not only in as-treated analysis, but also in the cohort of long-term use, we found that the heart failure risk was higher in febuxostat than in allopurinol users. However, there were some limitations to our study. First, unlike hospitalization of AMI or stroke which had been validated in previous studies, the coding of HF hospitalisations was not validated (41, 42). However, the Bureau of NHI regularly performs auditing reviews on a random sample of one per every 100 ambulatory claims and one per 20 inpatient claims quarterly and false reporting of diagnostic information results in a severe penalty from the Bureau. The coding validity of HF would be acceptable and misclassification of HF hospitalisations between febuxostat and allopurinol should be non-differential in this comparative study design. Second, the CV-related mortality was not been validated from death certificates, but the in-hospital mortality for AMI and stroke cases was validated, with a positive predictive value of 0.79 (43). Third, unmeasured confounding factors, such as uric acid level, blood pressure, BMI, and renal function, were not available in our study. Febuxostat was usually reimbursed when allopurinol or uricosuric treatment was ineffective in achieving target uric acid levels and in individuals who were intolerant to allopurinol. Gout patients who were resistant or intolerant to allopurinol or uricosuric treatment might be at higher risk of hyperuricemia than those who achieved effective control with allopurinol alone (44). Thus, we used a new-users design to minimize this confounding bias, as patients did not receive any study drug before 1 year on the index date. In addition, we used the rule-out sensitivity approach to estimate the extent of high levels of serum uric acid in association with allopurinol, febuxostat, and HF hospitalization risk. Further, we found it cannot possibly be strong enough confounders to explain the observed association obtained between allopurinol and febuxostat. Therefore, the effect of uric acid levels might be negligible in this study. Although we did not measure blood pressure or renal function, the baseline characteristics on renal disease and antihypertensive drugs were similar between febuxostat and allopurinol even before PS matching.

## Conclusion

Febuxostat use was associated with an increase in HF hospitalization risk compared to allopurinol use in gout patients, which was independent of low or high CV risk. Considering the risk of HF hospitalization would be highest in female gout patients with high CV risk who use febuxostat, HF monitoring is particularly warranted in these patients.

## Data availability statement

The original contributions presented in the study are included in the article/Supplementary material, further inquiries can be directed to the corresponding author.

## Ethics statement

This study was approved by the Institutional Review Board of the National Cheng Kung University Hospital (IRB certificate number B-EX-107-018). Written informed consent for participation was not required for this study in accordance with the national legislation and the institutional requirements.

## Author contributions

C-LC and C-TY carried out this population studies, participated in study design, interpretation of data, and drafting the manuscript. C-CS participated in the design of the study, performed the statistical analysis, and drafting the manuscript. C-HH participated in study design and drafting the manuscript. C-HL participated in interpretation of the data. Y-HY conceived of the study, participated in its design and coordination, and helped to draft the manuscript. All authors have read and approved the final manuscript.

## Funding

This work was supported by the Ministry of Science and Technology, Taiwan (MOST 104-2321-B-006−036 -) and National Cheng Kung University Hospital (NCKUH-10609002).

## Conflict of interest

The authors declare that the research was conducted in the absence of any commercial or financial relationships that could be construed as a potential conflict of interest.

## Publisher's note

All claims expressed in this article are solely those of the authors and do not necessarily represent those of their affiliated organizations, or those of the publisher, the editors and the reviewers. Any product that may be evaluated in this article, or claim that may be made by its manufacturer, is not guaranteed or endorsed by the publisher.
