# Supplementary material for: Sex difference in heart failure risk associated with febuxostat and allopurinol in gout patients
Source: Front Cardiovasc Med. 2022 Aug 11;9:891606. doi: 10.3389/fcvm.2022.891606 (PMC9403180; doi:10.3389/fcvm.2022.891606)
Supplement: Supplementary file 1 [file Data_Sheet_1.PDF]

Appendix

Supplemental table 1. Initial dose and length of medication exposure between allopurinol and febuxostat users.

Variable	Allopurinol	Febuxostat
Low CV risk group		
Initial dose (mg), median (p25, p75)	100 (100, 300)	40 (40, 80)
Length of medication exposure (days), mean (SD)	283 (247)	357 (372)
High CV risk group		
Initial dose (mg), median (p25, p75)	100 (100, 300)	40 (40, 80)
Length of medication exposure (days), mean (SD)	216 (366)	269 (322)

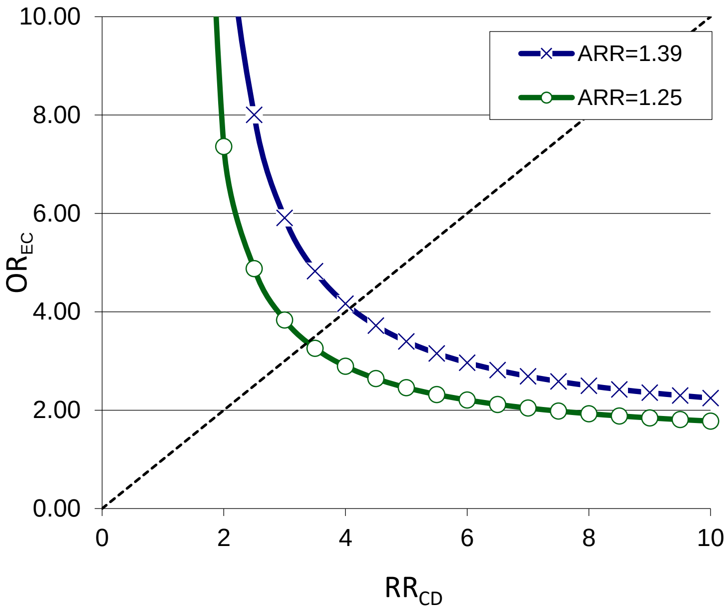

A

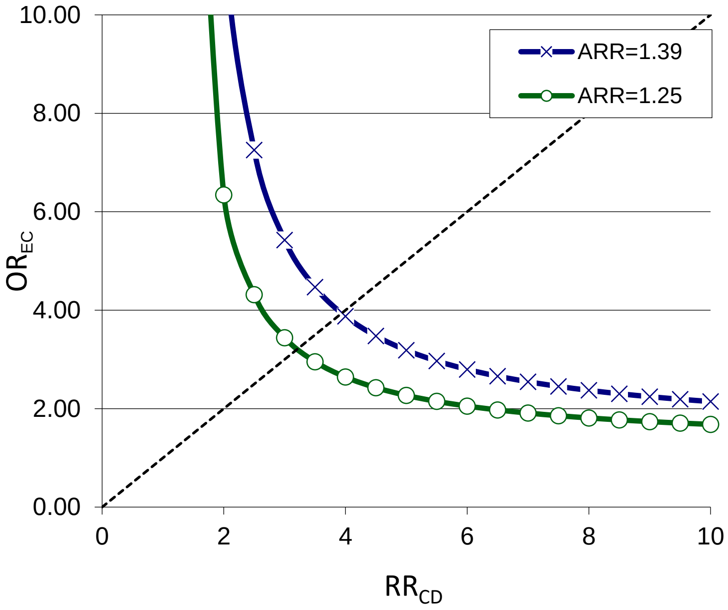

B

Supplemental Figure 1. Rule-out approach for exploring the effect of unmeasured confounding factors.

A. Effects of unmeasured confounding factors in patients without admission for AMI, HF, or stroke before the 3-year look-back period (low CV risk group)

B. Effects of unmeasured confounding factors in patients admitted for AMI, HF, or stroke prior to the 3-year look-back period (high CV risk group)

[a]OR_{EC}: Association between drug use category and confounder; RR_{CD}: association between confounders and disease outcome.

[b]Each curve splits the area into two areas: the upper right area represents all parameter combinations.
of OR_{EC} and RR_{CD}, which would create confounding by an unmeasured factor strong enough to move the point estimate of the apparent RR (ARR = 1.39) to the null (ARR=1) or even lower. Conversely, the area to the lower left represents all parameter combinations that are not able to move the ARR to the null.

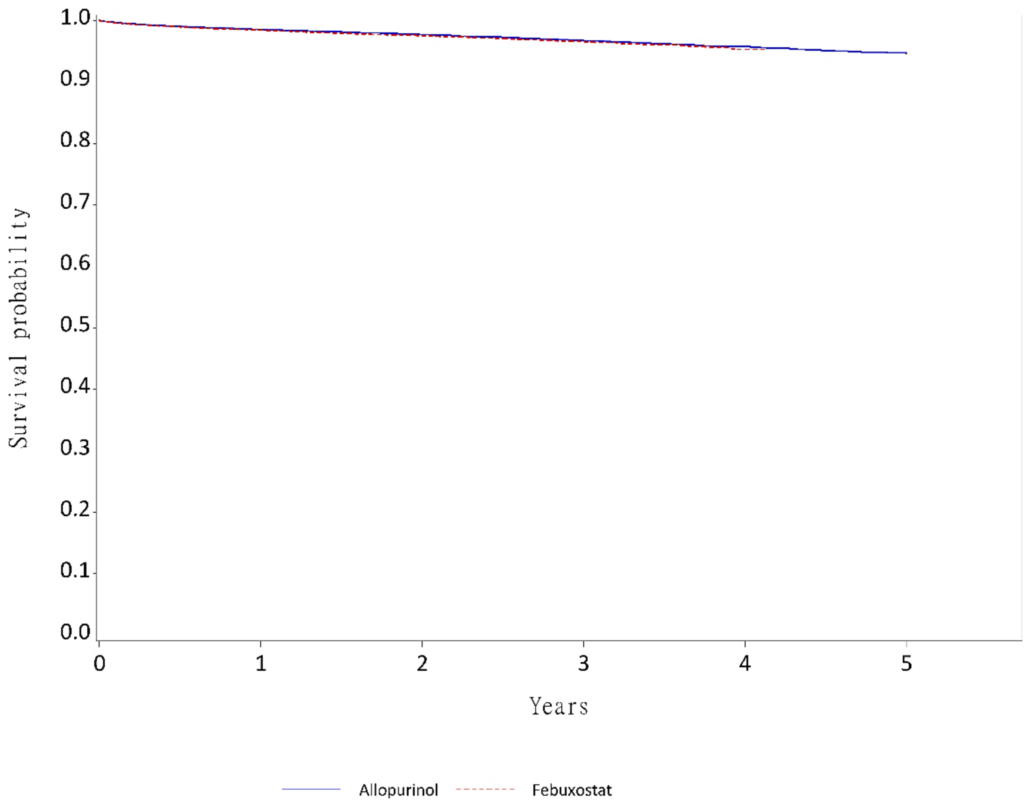
Survival probability
1.0
0.9
0.8
0.7
0.6
0.5
0.4
0.3
0.2
0.1
0.0
0
1
2
3
4
5
Years
Allopurinol
Febuxostat

A

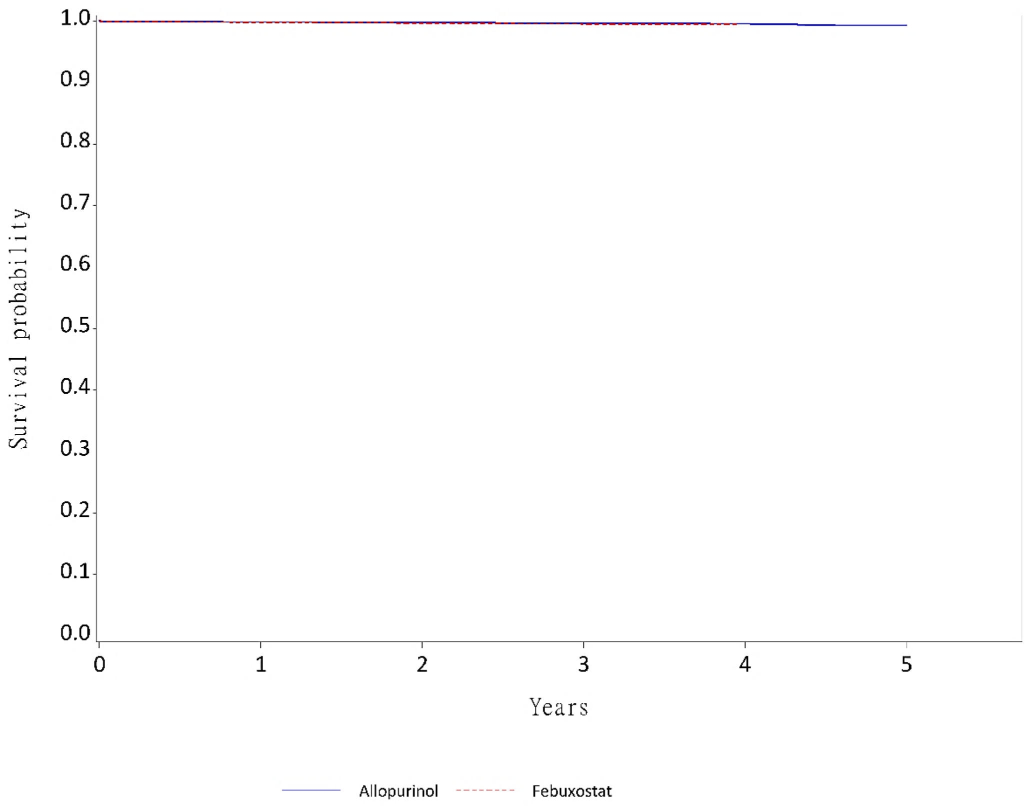
Survival probability
1.0
0.9
0.8
0.7
0.6
0.5
0.4
0.3
0.2
0.1
0.0
0
1
2
3
4
5
Years
Allopurinol
Febuxostat

B

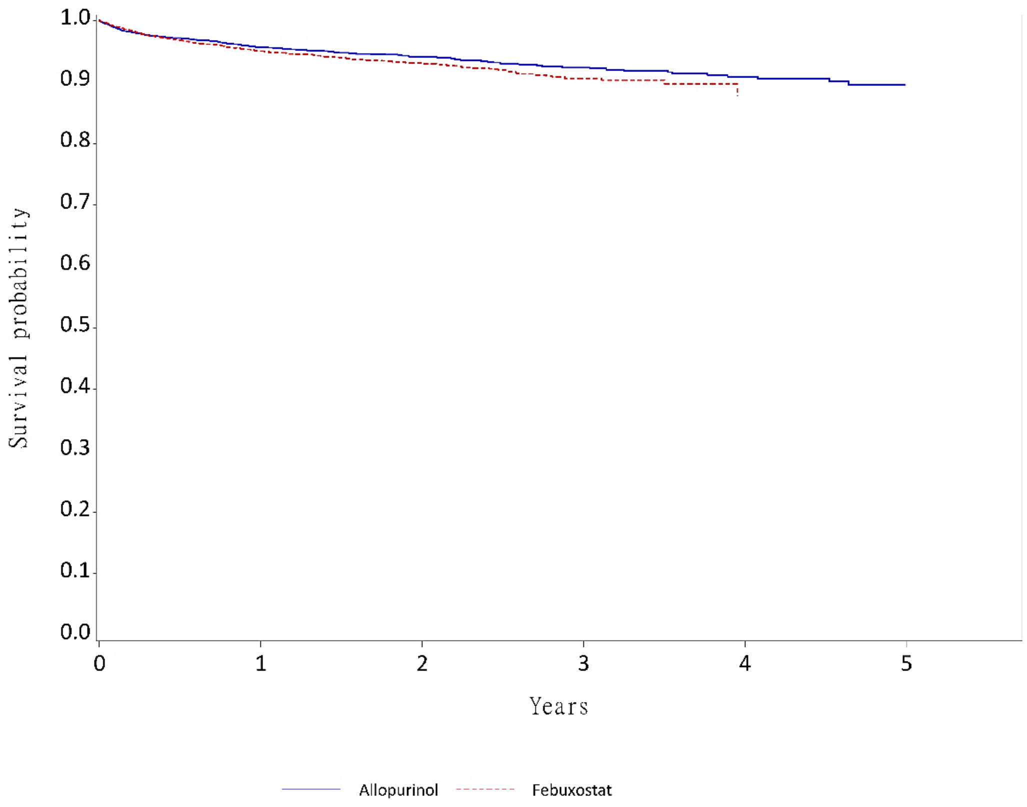

C

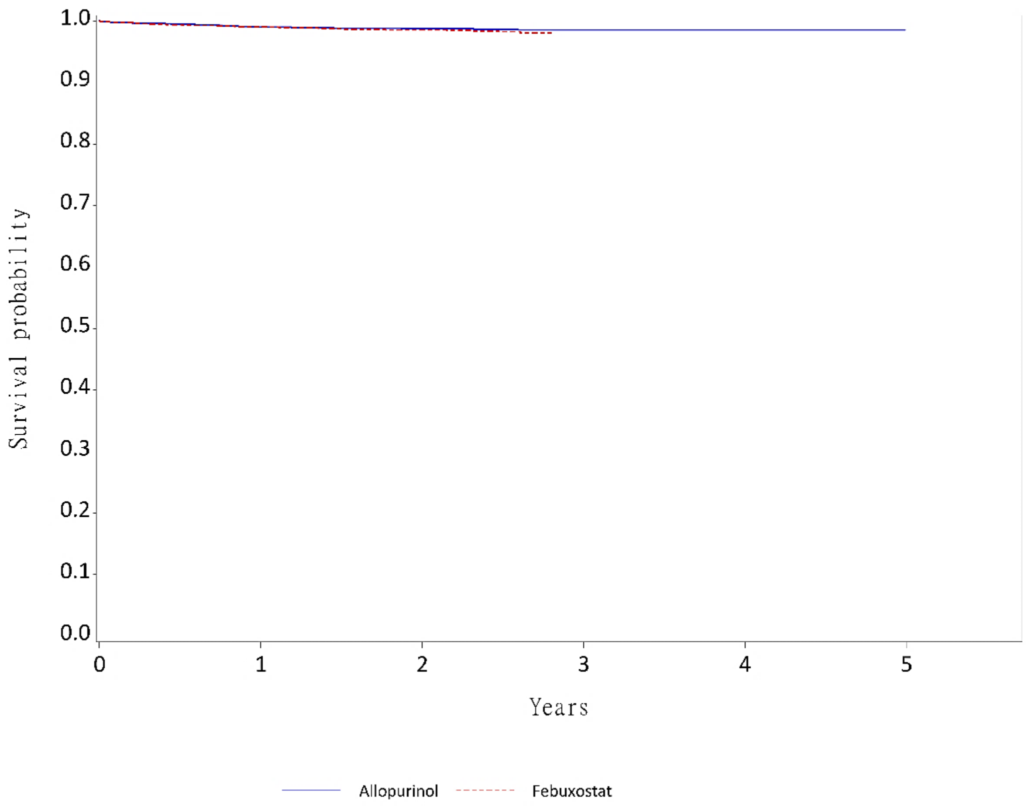

D

Supplemental Figure 2. The survival probability based on the number of years of medication use.

A. Survival function of time to all-cause mortality in patients without admission for AMI, HF, or stroke prior to the 3-year look-back period. (low CV risk group)

B. Survival function of time to cause CV death in patients without admission for AMI, HF, or stroke prior to the 3-year look-back period. (low CV risk group)

C. Survival function of time to all-cause mortality in patients admitted for AMI, HF, or stroke prior to the 3-year look-back period. (high CV risk group)

D. The survival function of time to cause CV death in patients admitted for AMI, HF, or stroke prior to the 3-year look-back period. (high CV risk group)

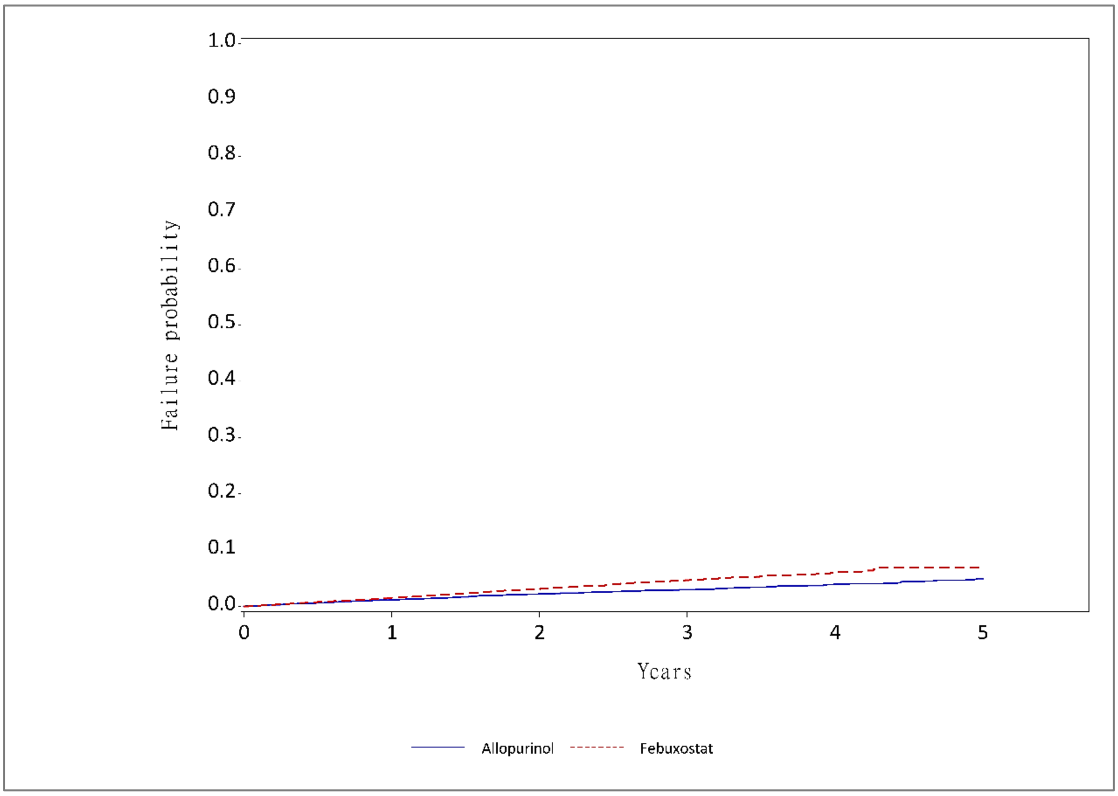

Supplemental figure 3. Kaplan-Meier of failure function for heart failure (as treated analysis)

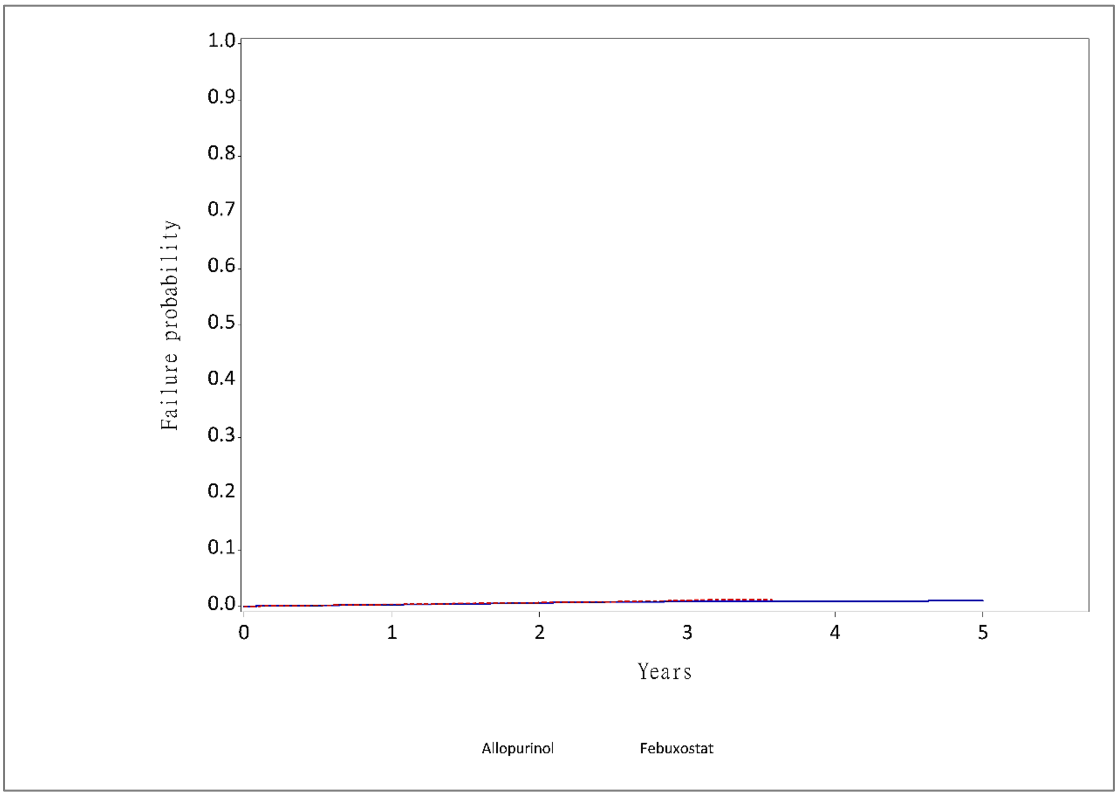

Supplemental figure 4. Kaplan-Meier of failure function for AMI (as treated analysis)

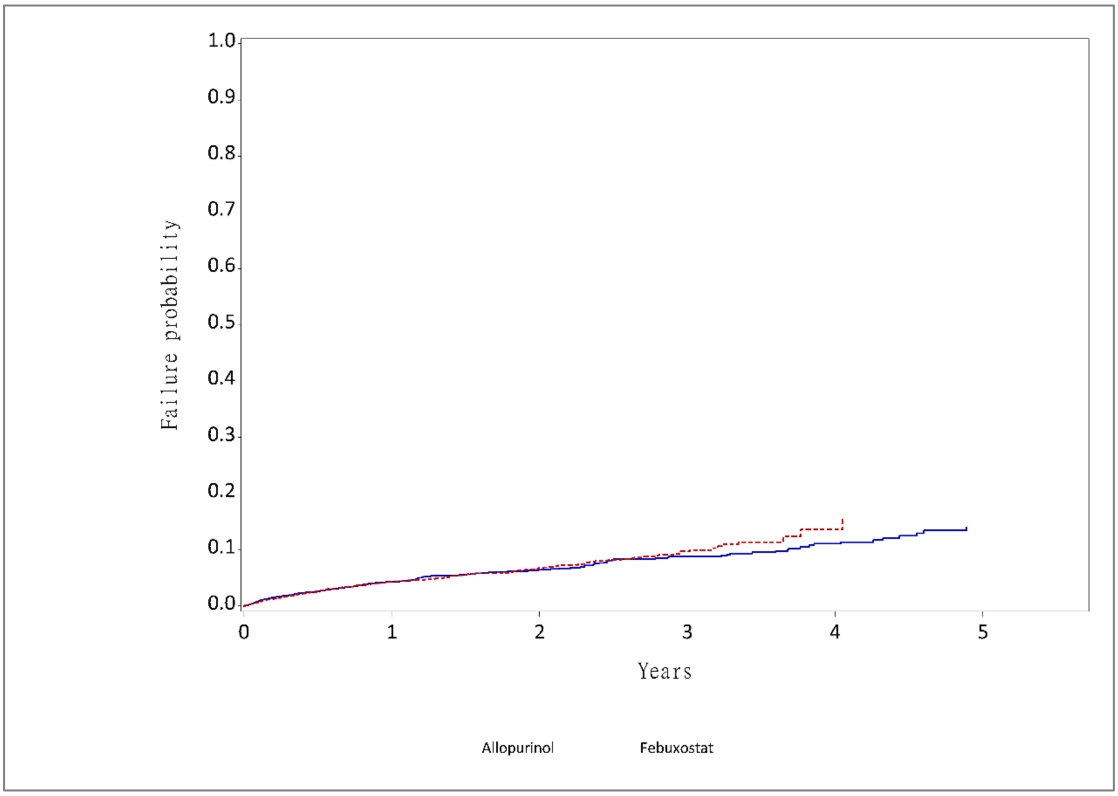

Supplemental figure 5. Kaplan-Meier of failure function for stroke (as treated analysis)

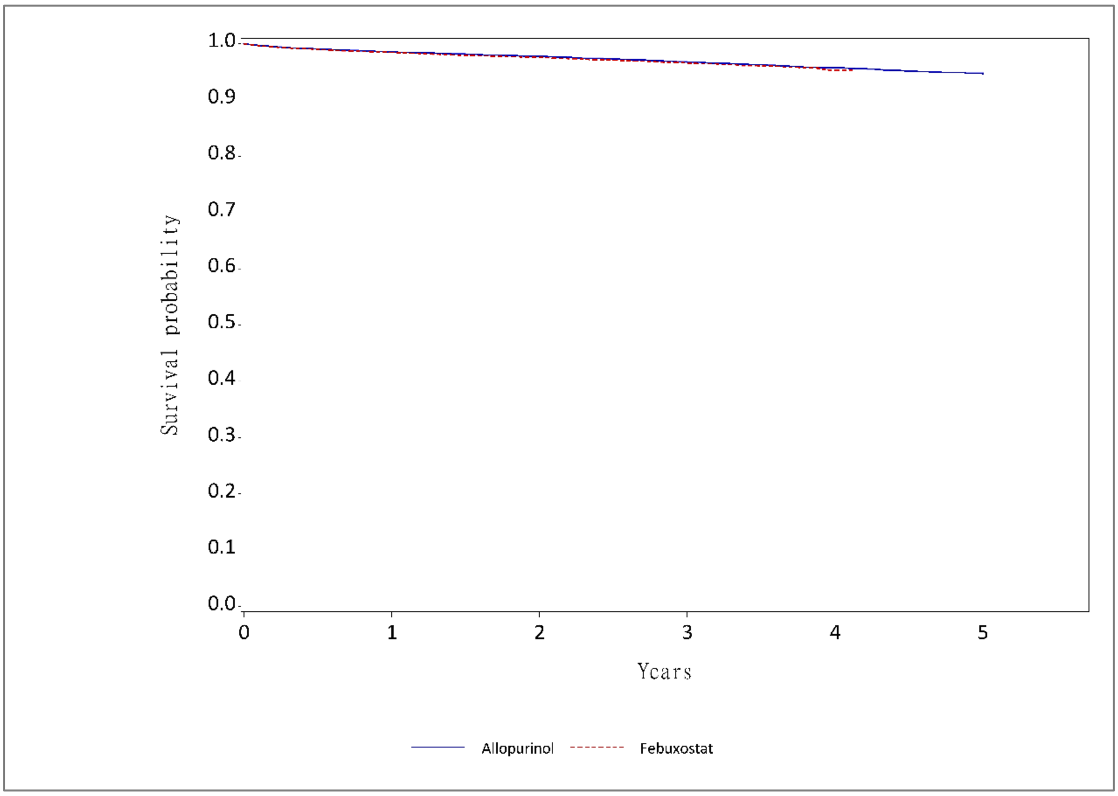

Supplemental figure 6. Kaplan-Meier of survival function for all-cause mortality (as treated analysis)

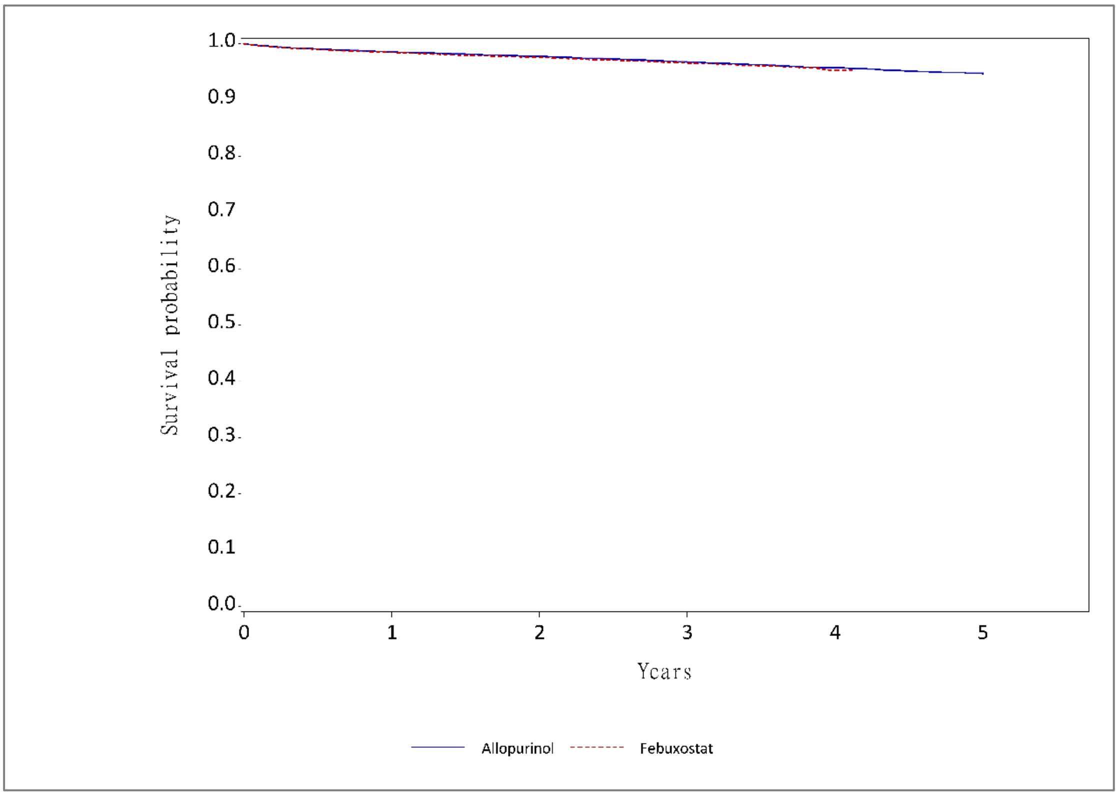

Supplemental figure 7. Kaplan-Meier of survival function for all-cause mortality (as treated analysis)

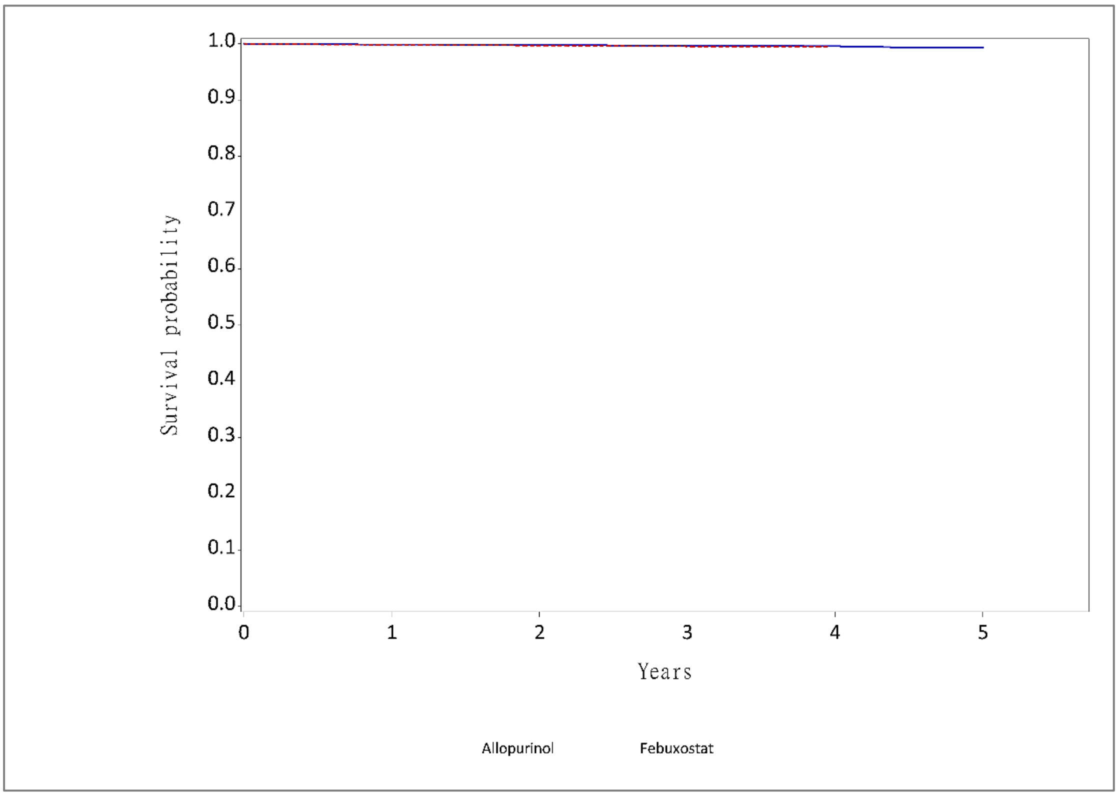

Supplemental figure 8. Kaplan-Meier of survival function for CV death (as treated analysis)
